# Revolutionizing African healthcare: harnessing co-creation, patient empowerment, and future thinking for patient outcomes

**DOI:** 10.3389/frhs.2026.1849774

**Published:** 2026-07-02

**Authors:** Kingsley Agyapong, Ebenezer Arthur Duncan, Samuel Affran

**Affiliations:** 1 Department of Management Studies Education, University of Skills Training and Entrepreneurial Development, Kumasi, Ghana; 2 Marketing, University of Professional Studies, Accra, Ghana; 3 Marketing and Entrepreneurship, University of Education Winneba, Winneba, Ghana

**Keywords:** co-creation, future thinking, patient-centered care, patient empowerment, patient outcomes

## Abstract

**Introduction:**

African healthcare systems continue to face poor patient outcomes, yet the role of co-creation in improving outcomes within low-resource settings remains poorly understood. Existing literature treats co-creation, patient empowerment, and future thinking as separate constructs and rarely examines the motivational and cognitive pathways linking them. Drawing on Self-Determination Theory (SDT) and Future-Oriented Theory (FOT), this study tests a dual-pathway model of co-creation in Ghanaian healthcare.

**Methods:**

A cross-sectional survey was conducted among 385 patients in Ghanaian hospitals in 2025. Structural equation modeling was used to assess relationships among co-creation, patient empowerment, future thinking, and patient outcomes, with empowerment and future thinking tested as parallel mediators.

**Results:**

Co-creation was positively associated with patient empowerment, future thinking, and patient outcomes. Both patient empowerment and future thinking partially mediated the relationship between co-creation and patient outcomes. The findings show co-creation improves outcomes primarily by building autonomy and competence, which then activate forward planning that guides health behavior.

**Discussion:**

This study extends SDT and FOT to African healthcare and reveals a previously untested dual-pathway mechanism in Ghana. Co-creation functions as an implementation-focused process rather than a decision-focused process in low-resource contexts, emphasizing patient adherence within resource constraints rather than treatment choice. For policy and management, co-creation initiatives should combine autonomy support with tools that help patients mentally simulate future health actions to improve outcomes.

## 1 Introduction

Healthcare systems worldwide are shifting away from traditional models to address rising patient demands and systemic inefficiencies (1, 2). Africa faces particularly severe pressures. Its growing population intensifies existing problems, including weak infrastructure, understaffed facilities, limited digitization, and fragmented health systems (3). Other barriers include restricted access (4), healthcare worker shortages and brain drain (5), low technological integration (6), and counterfeit medicines (7). These conditions highlight the need for a shift from top-down delivery to patient-centered designs that reflect cultural realities (8–10). Providers must build resilient, forward-looking approaches that prioritize patient perspectives while addressing equity gaps in health literacy and rural access (11).

Patient outcomes (POs)—defined as changes in health status, quality of life, and functional ability after healthcare interventions (12)—are central to healthcare quality. Standard measurement tools are essential for tracking these changes (13). Patient-centered strategies improve outcomes (14), and using patient-reported outcomes in planning and decision-making strengthens care quality (15). Advancing healthcare therefore requires building on existing knowledge and methods to achieve meaningful improvements (16).

Studies link co-creation (CC), patient empowerment (PE), and future thinking (FT) to better patient outcomes, but often treat them as separate concepts. Co-creation is associated with improved health outcomes, higher patient satisfaction, and stronger empowerment (17–21). Empowered patients demonstrate improved health behaviors, disease management, and autonomy (22, 23). In healthcare, future thinking is the cognitive ability to anticipate health states and plan self-management actions, using mental simulation of outcomes such as adherence or recovery to guide present behavior (24, 25). Future thinking supports positive health behaviors and overall wellbeing (26, 27).

Despite repeated calls for research (28–30), how co-creation, patient empowerment, and future thinking interact to shape patient outcomes in African healthcare remains unclear. Current studies in Africa rarely examine outcomes directly, focusing instead on delivery challenges (31), patient safety (32), measurement standardization across languages (33), and leadership quality (34). As a result, co-creation and patient empowerment are largely overlooked, and the motivational and cognitive pathways linking co-creation to outcomes—including autonomy support and future-oriented cognition—remain underexamined. The effects of future thinking, emerging technologies, and new healthcare models on outcomes are also unclear. While patient safety has been covered, core patient-centered principles like engagement, participation, and empowerment need deeper study. No framework yet integrates co-creation, patient empowerment, future thinking, and innovation to transform African healthcare. This leaves key questions: How do collaborative design and patient-centered care affect outcomes? What role does empowerment play? How can future thinking improve outcomes? Testing these interactions is essential to identify strategies for patient-centered care across diverse African settings.

This study develops a framework for healthcare design in Africa that integrates co-creation, patient empowerment, and future thinking to improve patient outcomes. Grounded in Self-Determination Theory (SDT) and Future-Oriented Theory (FOT), it uses quantitative data from 385 patients in Ghana. Results show positive associations among the variables, with patient empowerment and future thinking mediating the relationship between co-creation and patient outcomes. SDT explains how co-creation builds empowerment through autonomy support, while FOT explains how empowerment triggers future thinking to guide health behaviors and outcomes. The study extends both theories to the African context and offers practical insights for patient-centered care and health policy. It clarifies the collective impact of co-creation, empowerment, and future thinking on outcomes and maps their connections. The paper proceeds with literature review and hypotheses, methodology, results, discussion, and conclusion.

### 1.1 Literature review

#### 1.1.1 Background

African healthcare faces major constraints that limit both access and quality. Only 48% of the population has primary care access, leaving approximately 615 million without essential services (35). The region has 1.55 health workers per 1,000 people, below the WHO threshold (35). Other pressures include economic barriers to care (36), disparities in provision (37), inefficient systems (38), weak infrastructure investment (3), and fragile value co-creation processes. In Ghana, hierarchical provider–patient dynamics and short consultations limit patient empowerment (39). Low health literacy and rural access gaps further reduce patient competence, though the National Health Insurance Scheme (NHIS) and digital health tools are expanding agency (40, 41). Addressing these challenges requires patient-centered approaches (42, 43) and innovations such as technology-driven initiatives (31), stronger health systems, infrastructure investment, and public–private partnerships (44). Africa's pressing healthcare needs justify this study's focus.

#### 1.1.2 Theory: SDT and FOT

SDT explains human motivation through three basic needs: autonomy, competence, and relatedness (45, 46). Paternalistic care often fails because it thwarts these needs. While research shows co-creation and empowerment improve patient engagement, it rarely examines future thinking. This limits SDT's ability to explain how empowered patients maintain behavior change, since sustained action also requires cognitive planning. Linking SDT with co-creation, empowerment, and future thinking helps providers design interventions that support autonomy, competence, and relatedness while activating planning processes, leading to better outcomes.

##### 1.1.2.1 Future-oriented theory

Integrating SDT and FOT explains how co-creation affects patient outcomes, unlike past studies that treated these concepts separately (47–50). FOT argues that future perceptions shape current motivation and behavior (51). In this integrated model, SDT-based co-creation supports autonomy, which builds patient empowerment by meeting competence and self-determination needs. Empowerment then triggers future thinking, which links it to better outcomes. FOT bridges this gap by showing how future-oriented cognition translates present empowerment into health behavior change. SDT clarifies the motivational roots of empowerment, while FOT explains the cognitive pathway from empowerment to outcomes. Past models often stopped at empowerment, missing how prospection translates autonomy into sustained action. This integration shows how empowerment and future thinking connect co-creation to patient-centered outcomes.

##### 1.1.2.2 Patient outcomes

Patient outcomes reflect changes in health status, quality of life, and functional ability after healthcare interventions. They include clinical, functional, quality-of-life, and satisfaction measures (12, 52, 53). Although co-creation and patient empowerment are well studied (17, 20, 54), separate analyses miss a key point: Poor outcomes stem from two deficits. Motivational deficits need empowerment; planning deficits need future thinking. Linking these concepts shows how to design services that address both dimensions, thereby improving outcomes

##### 1.1.2.3 Co-creation

Co-creation in healthcare creates value when patients and providers collaborate, improving engagement, empowerment, health outcomes, and quality of life (55–61). Studies report benefits (18, 62) but do not explain mixed results. SDT provides a clarification: Collaboration without autonomy support fails to meet competence needs, making co-creation tokenistic. Using SDT and FOT, we can trace how co-creation affects outcomes by linking collaborative care to patient motivation and future planning.

##### 1.1.2.4 Patient empowerment

Patient empowerment encompasses health literacy, self-management, active participation, and communication (63). Empowered patients reduce costs, achieve better outcomes, and report higher satisfaction (64). They take active roles, make informed decisions with greater autonomy (65), and benefit from higher health literacy (63). Empowerment improves decision-making (23, 64), but FOT shows that it is not enough for sustained behavior change. Without prospection, patients cannot connect current choices to future health states. Using SDT and FOT, we can map how empowerment impacts outcomes by linking autonomy and competence to future-oriented motivation.

##### 1.1.2.5 Future thinking

Future thinking is the systematic process of envisioning, analyzing, and shaping potential futures (66). In healthcare, it refers to a patient's ability to anticipate, plan for, and mentally simulate future health states, treatment outcomes, and self-management actions. It includes prospection—projecting oneself into future health scenarios—and goal-directed planning toward desired outcomes. Examples include envisioning adherence consequences, anticipating recovery milestones, or planning lifestyle changes. This is health-specific mental time travel that guides current behavior, not generic optimism. System-level foresight (57, 67, 68) often overlooks patient-level prospection. Innovation fails if patients cannot mentally simulate adherence or recovery. However, research on how future thinking connects with co-creation and empowerment remains scarce. Linking these concepts shows how future thinking drives patient-centered, sustainable healthcare.

#### 1.1.3 Hypotheses development

##### 1.1.3.1 Co-creation and patient outcomes

Recognizing patients' needs and involving them in decisions produces more personalized, effective care, improving outcomes. Co-creation empowers patients to take active roles, raising engagement and linking to better health outcomes (57). Its collaborative nature builds trust and respect between patients and providers, leading to better outcomes (56). By respecting patient agency and autonomy, co-creation reduces power imbalances in traditional provider–patient relations, enabling more equitable and effective care (69).

H1: Co-creation positively affects patient outcomes.

##### 1.1.3.2 Co-Creation and patient empowerment

Co-creation enables collaborative engagement and informed decision-making between patients and providers (63). This raises autonomy, self-efficacy, and health literacy, strengthening patient empowerment. By building confidence, co-creation helps patients take active roles in care, supporting more equitable, patient-centered delivery.

H2: Co-creation has a significant positive effect on patient empowerment.

##### 1.1.3.3 Co-Creation and future thinking

Co-creation builds a culture of collaboration, shared responsibility, and proactive care (24, 67). Involving patients in care design produces personalized, preventive plans that help providers anticipate future health challenges (68). This links co-creation to prevention and better-quality outcomes through patient foresight.

H3: Co-creation has a positive effect on future thinking.

##### 1.1.3.4 Patient empowerment and patient outcomes

Empowerment raises self-efficacy, health literacy, and informed decision-making, which predict better outcomes (57, 63). Empowered patients manage disease more effectively, adhere to treatment, and demonstrate healthier behaviors, improving health outcomes (64). Empowerment also supports patient-centered care, reduces disparities, and increases health equity (63).

H4: Patient empowerment has a significant positive effect on patient outcomes.

##### 1.1.3.5 Future thinking and patient outcomes

Future thinking helps stakeholders anticipate and prepare for future health challenges (24). It drives anticipatory problem-solving, personalized care, innovation, and technology adoption (57, 67). Future thinking also increases patient empowerment, collaboration, adaptability, and sustainability, thereby improving outcomes, satisfaction, and resource efficiency (25).

H5: Future thinking has a positive effect on patient outcomes.

##### 1.1.3.6 Co-creation and patient outcomes through patient empowerment

Co-creation builds patient empowerment, which in turn influences outcomes. Involving patients in care design and delivery raises autonomy, self-efficacy, and confidence, thereby improving health outcomes. Self-determination theory supports this mediation, showing why empowerment links co-creation to outcomes.

H6: Patient empowerment mediates the relationship between co-creation and patient outcomes.

##### 1.1.3.7 Co-creation and patient outcomes through future thinking

Co-creation increases future thinking, which then influences outcomes. Co-creation helps providers and patients anticipate and prepare for future health challenges. Future-oriented theory supports this mediation, showing why prospection links co-creation to outcomes. Evidence demonstrates that future thinking improves patient outcomes (70, 71).

H7: Future thinking mediates the relationship between co-creation and patient outcomes.

##### 1.1.3.8 Conceptual framework

**Figure 1:**
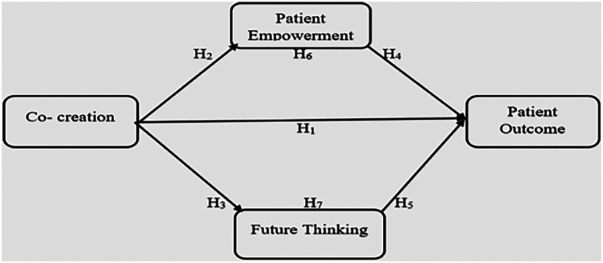
Conceptual framework. Source: Study's conceptualization (2025).

We developed a conceptual framework for the African healthcare context, shown in Figure 1. Co-creation is hypothesized to have a direct effect on patient outcomes (H1), and to increase both patient empowerment (H2) and future thinking (H3). In turn, empowerment (H4) and future thinking (H5) are hypothesized to improve patient outcomes. Thus, co-creation affects outcomes indirectly through empowerment (H6) and future thinking (H7). The framework also allows for reciprocal links. Empowerment may feed back into co-creation, creating a reinforcing loop. Future thinking may also shape co-creation in an iterative process. This study tests both the direct and indirect effects. Future work could examine moderation or bidirectional causality.

## 2 Methodology

### 2.1 Context and target population

The target population consists of patients aged 18+ in Ghanaian hospitals, both public and private. Ghana is a West African country with approximately 31 million people (94). Ghana has improved its healthcare system, but delivery, patient satisfaction, and health outcomes remain challenges (72). Patients in Ghanaian hospitals are well positioned to report their perceptions of co-creation, empowerment, and future thinking in care.

### 2.2 Sampling and sample size

The number of patients who visited public hospitals in Ghana during data collection was unknown. We used Cochran's formula to determine sample size. The formula is suitable for large populations and unknown population sizes (73). It is also widely used in survey research and provides reliable sample estimates (74).

$$n=(Z\;\hat{\;}2,*\;p\;*\;q)/E\;\hat{\;}2$$

where n = sample size, Z = Z-score (e.g., 1.96 for 95 percent confidence level), p = proportion of interest (we used 0.5 as a conservative estimate to maximize sample size), q = 1 − p, and E = margin of error (0.05 for 5% margin of error).

Substituting these values into the formula, the required sample size was calculated as:

$$n=(1.96\;\hat{\;}2*0.5*0.5)/0.05\hat{\;}2$$

where *n* = 384.16.

Cochran's formula indicated a target sample of 385 patients. Multi-stage sampling was used (75). Greater Accra was purposively chosen as the study site due to its concentration of healthcare facilities and diverse population (76). Major hospitals in the region were listed, assigned numbers, and three hospitals were selected using a random number generator (77). From 1 to 29 November 2025, every third patient in outpatient waiting areas who met the inclusion criteria was approached: aged ≥18, able to provide oral consent, and receiving care that day. Of 520 eligible patients approached, 401 participated and 385 provided complete responses. The response rate was 77.1%. The sampling frame included three urban hospitals in Greater Accra and did not constitute a simple random sample of all Ghanaian patients (78).

### 2.3 Data collection instrument

Data on co-creation, patient empowerment, future thinking, and patient outcomes were collected using a 28-item questionnaire with a five-point Likert scale. Items were adapted from four established scales: the Co-Creation Scale (61), Patient Empowerment Scale (79), Future Orientation Scale (80), and Patient Outcomes Scale (81). The questionnaire was pilot-tested with Ghanaian patients to check cultural relevance and validity. Trained research assistants interpreted questions in local languages for participants needing help. Face validity, content validity, and pre-testing supported instrument validity (82). Procedural and statistical techniques addressed common method bias (83).

### 2.4 Complying with ethics of experimentation

Ethical clearance was obtained from the institutional review board. The study was non-invasive and anonymous. Oral informed consent was obtained from all participants before questionnaire administration. The study purpose, voluntary participation, and right to withdraw without penalty were explained. No personal identifiers were collected.

## 3 Results

### 3.1 Demographic characteristics of the respondents

The demographic characteristics of the respondents are presented in Table 1.

**Table 1:** Demographic profile of respondents.

Demographic characteristics	Range	Frequency (n)	Percentage
Age	18–24	77	20
	25–34	116	30
	35–44	96	25
	45+	96	25
Education	High school	77	20
	Diploma	96	25
	Bachelor's degree	116	30
	Postgraduate degree	96	25
Gender	Male	173	45
	Female	193	50
	Others	19	5
Annual household income	GH¢5,000–10,000	77	20
	GH¢10,001–20,000	96	25
	GH¢20,001–50,000	77	20
	GH¢50,001–100,000	70	18
	Above GH¢100,000	65	17
Residence	Urban	154	40
	Semi-urban	135	35
	Rural	77	20
	Peri-urban	19	5

Source: Primary data (2025).

Demographic results show respondent characteristics and highlight trends in perceptions of co-creation, patient empowerment, and future thinking in healthcare. Respondent distribution across education, income, and residence indicates a diverse sample, which helps identify disparities and improvement opportunities in healthcare services.

### 3.2 Result

Relationships between variables were examined using Partial Least Squares Structural Equation Modeling (PLS-SEM 4.0). PLS-SEM models relationships between latent and observed variables. It handles complex relationships and dependencies among model variables (84). SEM involves two procedures: measurement analysis, which tests links between latent variables and indicators, and structural modeling, which tests links between latent variables and verifies hypotheses (85).

#### 3.2.1 Measurement model assessment

**Figure 2:**
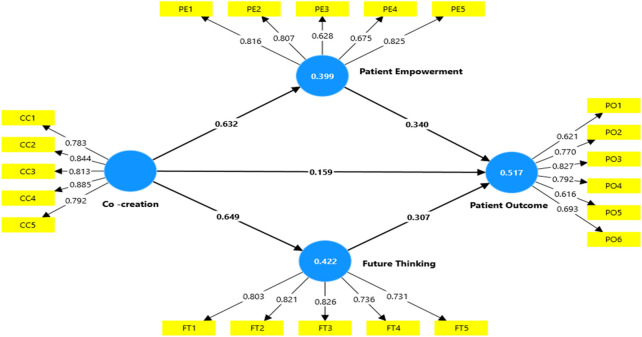
Measurement model output. Source: Primary data (2025).

The measurement model was assessed using outer loadings, construct reliability, and construct validity (85, 86). Table 2 shows that outer loadings exceeded 0.7, indicating that items appropriately reflected their constructs (95). Construct reliability, measured by Cronbach's Alpha and Composite Reliability, was above 0.7 for all constructs, showing consistent results (87, 88). Convergent validity was supported as average variances extracted (AVEs) for CC, PO, FT, and PE exceeded 0.5 (96). Discriminant validity was confirmed with heterotrait–monotrait results (HTMT) values below 0.85 (88), as shown in Table 3 and Figure 2.

**Table 2:** Reliability and validity results.

Items	Outer loading values	Cronbach's alpha	Composite reliability (rho a)	AVE
Co-Creation		0.881	0.885	0.679
CC1	0.783			
CC2	0.844			
CC3	0.813			
CC4	0.885			
CC5	0.792			
Patient outcomes		0.816	0.832	0.525
PO1	0.621			
PO2	0.770			
PO3	0.827			
PO4	0.792			
PO5	0.616			
PO6	0.693			
Future thinking		0.843	0.854	0.615
FT1	0.803			
FT2	0.821			
FT3	0.826			
FT4	0.736			
FT5	0.731			
Patient empowerment		0.808	0.867	0.569
PE1	0.816			
PE2	0.807			
PE3	0.628			
PE4	0.675			
PE5	0.823			

Source: Primary data (2025).

**Table 3:** Heterotrait – Monotrait Results (HTMT) and Fornell – Larcker Criterion.

Construct	CC	FT	PE	PO	1	2	3	4
Co-creation (1)					0.773			
Future thinking (2)	0.750				0.599	0.735		
Patient empowerment (3)	0.734	0.611			0.488	0.649	0.532	
Patient outcomes (4)	0.667	0.782	0.712		0.574	0.54	0.661	0.724

Source: Primary data (2025).

#### 3.2.2 Indicator reliability

Table 2 reports outer loadings for each indicator. Loadings above 0.70 are required to confirm that constructs explain indicator variance (89). All outer loadings in Table 2 exceeded 0.70, signifying indicator reliability.

Table 3 presents HTMT ratios and Fornell–Larcker results used to assess discriminant validity. HTMT ratios compare between-construct correlations to within-construct correlations. Values below 0.85 indicate distinct constructs (90).

HTMT values were 0.750 between future thinking and co-creation, 0.734 between patient empowerment and co-creation, and 0.611 between patient empowerment and future thinking. All values were below 0.85, confirming discriminant validity (90). Fornell–Larcker results in Table 3 also show that the square root of each construct's AVE exceeded correlations with other constructs, indicating that the constructs are distinct. Together, HTMT and Fornell–Larcker results confirm discriminant validity for CC, FT, PE, and PO. Distinct constructs ensure that the model measures separate aspects. Figure 2 presents the measurement model, showing relationships between latent constructs and indicators. Results support the reliability and validity of the measurement instruments.

#### 3.2.3 Structural model evaluation

The structural model was tested after measurement model validation to examine relationships among constructs, assess the coefficient of determination (R^2^), and evaluate predictive accuracy. R^2^ quantifies how independent variables explain variance in dependent variables. Table 4 reports the R^2^ values for the three endogenous constructs: 0.422 for future thinking, 0.399 for patient empowerment, and 0.517 for patient outcomes. These indicate that 42.2%, 39.9%, and 51.7% of variance is explained, respectively, showing moderate to substantial explanatory power (89). These results indicate that co-creation influences patient-centered healthcare experiences in Ghanaian hospitals. The 51.7% R^2^ for patient outcomes shows that collaborative provider–patient interactions substantially affect perceived healthcare effectiveness, treatment engagement, and wellbeing. Patient outcomes in African healthcare settings are shaped by both clinical interventions and participatory practices that strengthen trust, involvement, and shared decision-making.

**Table 4:** Predictive relevance of model.

Constructs	VIF	f^2^	R^2^	Adjusted R^2^	Q^2^
CC -> FT	1.000	0.729			
CC -> PE	1.122	0.664	0.422 (FT)	0.421	0.420
CC -> PO	1.920	0.027	0.399 (PE)	0.399	0.398
FT -> PO	2.358	0.083	0.517 (PO)	0.516	0.328
PE -> PO	2.269	0.106			

Source: Primary data (2025). Magnitude effect sizes (f^2^) of 0.02, 0.15, and 0.35 indicate small, medium, and significant effects, respectively (97). CC, co-creation, FT, future thinking, PE, patient empowerment, PO, patient outcomes.

The Stone–Geisser Q^2^ was used to assess predictive relevance. Q^2^ values must exceed zero to indicate predictive accuracy (90). Table 4 shows Q^2^ values of 0.420 for future thinking, 0.398 for patient empowerment, and 0.328 for patient outcomes. Positive Q^2^ values confirm that the model has predictive relevance for FT, PE, and PO. This supports the model's out-of-sample predictive capability and applicability to patient-centered healthcare dynamics in emerging systems such as Ghana.

Table 4 also reports f^2^ effect sizes, R^2^, Q^2^, and variance inflation factor (VIF) values for the structural model. f^2^ values range from small to large, indicating varying substantive impacts across paths (Cohen, 1988). Co-creation shows large effects on future thinking (f^2^ = 0.729) and patient empowerment (f^2^ = 0.664), but a small direct effect on patient outcomes (f^2^ = 0.027). This pattern indicates that co-creation influences patient outcomes primarily through empowerment and future thinking rather than directly. VIF values from 1.000 to 2.358 are below 5.0, confirming no multicollinearity (89). Positive Q^2^ values support predictive relevance.

#### 3.2.4 Path coefficient

Path coefficients were assessed using *β*-values for direction, t-values, and *p*-values for significance (91). Paths with *p* < 0.05 and t > 1.96 are statistically significant (89). Table 5 shows that hypotheses H1 through H7 were supported. All hypothesized relationships were significant. The strong path from co-creation to patient empowerment indicates that active patient participation increases confidence, autonomy, and treatment ownership. The significant effect of future thinking on patient outcomes shows that future-oriented patients engage more in preventive behaviors and treatment adherence. These results support shifting from provider-dominated systems toward collaborative, patient-centered models in African healthcare.

**Table 5:** Hypotheses testing.

Structural relationship	Hypotheses	Standardized beta (*Β*)	T-statistics (t-Value > 1.96)	*p*-values	Status of the hypothesis
Direct effect					
CC → PO	H1	0.159	6.159	0.000	Supported
Direct with mediator					
CC → PE	H2	0.632	35.581	0.000	Supported
CC → FT	H3	0.649	39.757	0.000	Supported
PE → PO	H4	0.340	12.344	0.000	Supported
FT → PO	H5	0.370	16.046	0.000	Supported
Indirect effect					
CC → PE → PO	H6	0.215	11.369	0.000	Supported
CC → FT → PO	H7	0.199	11.226	0.000	Supported

Source: Primary Data (2025).

#### 3.2.5 Model fitness

**Figure 3:**
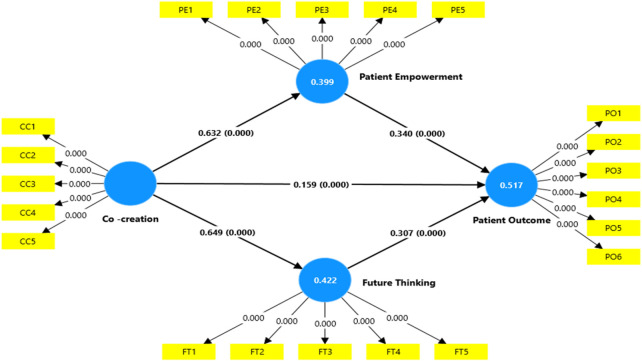
Structural model output. Source: Primary data (2025).

Model fit was assessed using standardized root mean square residual (SRMR), d_ULS, d_G, chi-square, and normed fit index (NFI) (89). Thresholds for acceptable fit are SRMR <0.08, d_ULS <0.08, d_G <0.10, non-significant chi-square *p* >0.05, and NFI values close to 1 (85). Table 6 shows that the model meets these criteria. Acceptable SRMR, d_ULS, d_G, and NFI values indicate that the model adequately represents the data and supports the hypothesized relationships. This confirms model robustness and applicability to healthcare management in African systems. Figure 3 presents the structural model with path coefficients between latent constructs.

**Table 6:** Summary of model Fit.

Fit index	Saturated model	Estimated model
SRMR	0.079	0.077
d_ULS	0.072	0.078
d_G	0.088	0.093
Chi-square	728.786	697.338
NFI	0.947	0.958

Source: Primary data (2025).

## 4 Discussion

The findings clarify connections among co-creation, patient empowerment, future thinking, and patient outcomes. Viewed through SDT (45) and Future Time Perspective (FTP) (51), this research identifies mechanisms underlying these relationships and shows how context shapes them. In line with SDT's claim that social contexts enable or hinder psychological needs, results indicate that Ghana's health system environment conditions how co-creation fosters empowerment. Structural enablers such as National Health Insurance Scheme coverage and digital health tools (40, 41) support autonomy and competence by reducing cost barriers and expanding health information access. Conversely, hierarchical provider–patient dynamics and consultations under 10 min (39) can undermine autonomy, while rural access gaps and low health literacy limit competence. Thus, whether co-creation meets patients’ needs for autonomy, competence, and relatedness depends on these system supports and barriers. This contextual dependence suggests that the co-creation–empowerment link may be stronger for patients with higher education, higher income, or urban residence, who use co-creation opportunities more effectively, and weaker for those with financial or geographic constraints. Although demographic data were collected, subgroup differences were not tested.

Building on this contextual framing, H1 examined the direct link between co-creation and patient outcomes. H1 was supported, showing co-creation positively associated with patient outcomes. This finding aligns with literature on patient-centered care (69, 92). However, the relationship appears weaker in Ghana than in Western settings, likely because structural friction impedes implementation. While NHIS coverage reduces catastrophic expenditure (40), frequent drug stockouts and “pay-before-service” practices mean that jointly developed care plans often stall at access. Consequently, this association indicates that co-creation improves outcomes only when institutional mechanisms enable shared decisions. For SDT, this shows that autonomy satisfaction is necessary but insufficient; competence also requires material resources, not just psychological support. A competing explanation is measurement scope: Outcome indicators emphasized clinical metrics, whereas Ghanaian patients often prioritize respectful treatment over biomedical endpoints (39). This implies that SDT requires a “resource contingency” boundary condition for low- and middle-income countries, where psychological need satisfaction is limited by service delivery capacity.

Turning to the psychological mechanism, H2 posited a positive effect of co-creation on patient empowerment. The data supported H2, showing co-creation positively associated with patient empowerment, consistent with SDT's premise that involvement fulfills autonomy and competence needs (46, 63). The strength of this association is notable and differs from prior Western studies. This difference may reflect a “low baseline effect”: In Ghana's paternalistic system, where consultations average under 10 min (39), even minimal invitations to contribute can produce substantial empowerment gains. Nevertheless, this association is likely limited by sociocultural factors. In rural settings with low health literacy and family elders mediating clinical decisions, co-creation could undermine autonomy if patients feel pressured to show agency they lack (41). For theory, SDT assumes volitional engagement. In collectivist low- and middle-income countries (LMIC) contexts, relatedness may outweigh autonomy, indicating that SDT should specify when autonomy is culturally valued vs. imposed. Future theoretical work should therefore include “constrained autonomy”—empowerment under structural limits.

In a related vein, H3 examined whether co-creation fosters future thinking. H3 was supported, showing co-creation positively associated with future thinking, consistent with FTP's claim that collaboration extends temporal horizons (24, 51). An unexpected pattern emerged: Future thinking had a stronger association with outcomes than co-creation's direct association. Within Ghana's healthcare landscape, marked by rising chronic disease burdens but weak preventive infrastructure (68), co-creation's main utility may be cognitive, helping patients envision adherence pathways despite system deficiencies. This cognitive function is supported by recent digital interventions, such as NHIS-linked SMS appointment reminders (41), which were previously absent. Without such tools, co-creation might not activate FTP. This suggests that FTP assumes temporal stability. In settings with NHIS reimbursement arrears and drug uncertainty, “future thinking” may reflect present-biased coping rather than long-term planning. Accordingly, future theoretical work should disaggregate FTP dimensions and introduce a “scarcity moderator.”

Further, H4 tested the empowerment–outcomes link. H4 was supported, showing empowerment positively associated with outcomes, consistent with SDT (45) and LMIC evidence (12, 52, 57). Although this aligns with existing literature, the effect size is smaller than Western meta-analyses report. This difference highlights an “empowerment-capability gap.” In Ghana, patients may feel autonomous in selecting treatments, but NHIS non-coverage or stockouts prevent choice realization, limiting outcome improvements. Here, Sen's capability approach (93) provides a competing explanation: Autonomy must translate into real freedoms to improve health. Consequently, SDT and capability perspectives should be integrated for LMICs, recognizing that empowerment improves outcomes only when structural capabilities exist.

Similarly, H5 assessed future thinking's impact on outcomes. H5 was supported, showing future thinking positively associated with outcomes, consistent with FTP (51, 71). Contrary to some Western models where empowerment predominates (92), future thinking had a stronger direct association with outcomes than empowerment in this study. In contexts of frequent system shocks—drug shortages and referral delays—patients who mentally simulate adherence strategies may better navigate barriers, positioning FTP as a form of resilience. A negative finding warranting explanation is the untested possibility that future thinking becomes maladaptive under extreme scarcity, producing anxiety rather than planning. The literature remains silent on this boundary, suggesting that FTP requires elaboration for precarious environments.

Finally, the mediation hypotheses clarify how co-creation operates. Both H6 and H7 were supported: Patient empowerment and future thinking partially mediated the relationship between co-creation and patient outcomes. This confirms SDT's account that co-creation functions through need satisfaction rather than directly (45), consistent with Akter et al. (69) and Epstein et al. (70). The indirect effects exceeded the direct effect, indicating that co-creation's value in Ghana is primarily psychological—building agency and foresight—rather than changing clinical protocols. Structurally, this reflects formulary constraints limiting physician discretion; patients co-create how to adhere, not what treatment to receive. For theory, SDT's mediation chain holds, yet autonomy content differs across contexts. Whereas Western models assume choice among therapeutic options, LMIC models may emphasize choice in execution. Future theory development should therefore distinguish “decisional co-creation” from “implementational co-creation.”

These patterns suggest two critical considerations. First, a competing explanation for the moderate explained variance is the omission of structural variables—such as NHIS status, facility level, and waiting time—which likely condition these pathways. The co-creation–empowerment link may be weaker for low-education, rural patients who were underrepresented in this urban sample. Second, for practice, hospital managers should pair co-creation initiatives with NHIS education, real-time drug availability information, and literacy-adjusted communication; otherwise, co-creation risks becoming “empowerment theater” rather than substantive patient involvement.

### 4.1 Theoretical implications

The study's findings have key theoretical implications for three frameworks: SDT, FTP, and Co-Creation. Empirical validation of SDT confirms that autonomy support fosters patient empowerment, which then improves health outcomes. This supports SDT's postulates that autonomy, competence, and relatedness drive intrinsic motivation and optimal functioning.

However, this study extends SDT by specifying a “resource contingency” boundary condition for LMICs: Psychological need satisfaction is limited by material capability and decisional space. In Ghana, autonomy without drug availability or NHIS coverage does not improve outcomes, showing that competence requires structural plus psychological supports. The study's contribution to FTP demonstrates that future thinking mediates the co-creation–outcomes link. This finding supports the FTP framework, showing that future-oriented perspectives shape patient behavior and outcomes. Yet findings also suggest that FTP needs a “scarcity moderator” for precarious environments: Future thinking functions as resilience under moderate uncertainty but may become maladaptive when futures seem uncontrollable due to system shocks such as NHIS arrears and drug stockouts.

Furthermore, the study extends co-creation by showing that patient empowerment and future thinking are central to co-creation initiatives. Clarifying the interplay among co-creation, empowerment, and future thinking, this research reveals mechanisms underlying effective co-creation in healthcare. In particular, results reframe co-creation in LMICs as “implementational” rather than “decisional”: Patients co-create how to adhere within formulary and resource constraints, not what treatment to receive. This distinction challenges universal applications of co-creation and SDT. Integrating Sen's (93) capability approach, SDT must include structural capabilities to explain outcome variance in resource-constrained systems, moving beyond purely psychological need satisfaction.

#### 4.1.1 Managerial implications

The study's findings have key implications for healthcare management and delivery. Healthcare managers should prioritize patient empowerment and future-oriented thinking in co-creation initiatives to advance patient-centered care and improve health outcomes. Using co-creation strategies, managers can facilitate patient empowerment, future thinking, and collaboration, ultimately enhancing outcomes and patient satisfaction. Furthermore, integrating future thinking and patient empowerment into healthcare delivery can promote proactive health behaviors and informed decision-making, demonstrating the value of a forward-thinking approach in healthcare management. These findings indicate that healthcare managers should reorient strategies toward patient-centered care, co-creation, and future-oriented thinking to improve delivery and outcomes.

#### 4.1.2 Policy implications

If the co-creation–outcomes association holds causally, policy should formalize shared decision-making protocols and provider training to support autonomy, expand digital health tools to strengthen competence, and engage caregivers while safeguarding patient autonomy. Equitable implementation requires targeted health literacy support for patients with lower education, income, or rural residence, who may gain less from co-creation.

### 4.2 Limitations

The findings should be interpreted in light of several limitations. First, the quantitative design may not fully capture complex dynamics among co-creation, empowerment, future thinking, and patient outcomes, potentially missing contextual influences. It also cannot explain why empowerment levels vary across patients, as qualitative interviews were not conducted to capture lived experiences, cultural views of autonomy, or provider–patient interaction nuances. Second, while grounded in Ghana's health system, this study did not examine how family or social contexts shape self-determination. In Ghanaian settings, health decisions are often mediated by family and communal norms that can strengthen relatedness or limit autonomy. Third, despite multi-stage sampling, purposively selecting Greater Accra and sampling hospitals/patients within it may limit generalizability. The sampling frame covered three urban hospitals in Greater Accra and therefore does not constitute simple random sampling of all Ghanaian patients. Results may not extend to rural settings, primary-level facilities, or other regions where infrastructure, health literacy, and provider–patient dynamics differ. Fourth, although data on education, income, and residence were collected (Table 1), whether the co-creation–empowerment link differs across these subgroups was not tested. Fifth, reliance on self-reported data may introduce social desirability and recall bias. Sixth, the cross-sectional design precludes causal inference. Finally, findings may not extend beyond this healthcare context.

### 4.3 Further studies

Future research should prioritize the following directions. Longitudinal studies can establish causal links between co-creation, patient empowerment, future thinking, and patient outcomes. Mixed-methods approaches can capture the complexity of these relationships by integrating quantitative and qualitative data, with interviews specifically examining why some patients gain more empowerment from co-creation than others. Testing whether the co-creation–empowerment link differs by education, income, or urban/rural status can clarify for whom co-creation works best. Examining diverse populations and healthcare settings can improve generalizability. Intervention studies can design and evaluate initiatives promoting co-creation, patient empowerment, and future thinking, while comparative studies can assess alternative co-creation strategies. These directions can produce a fuller understanding of co-creation, patient empowerment, and future thinking in healthcare.

## 5 Conclusion

In conclusion, this study clarifies the links among co-creation, patient empowerment, future thinking, and patient outcomes in Ghanaian hospitals. The findings show that co-creation, patient empowerment, and future thinking improve patient outcomes, opening avenues for patient-centered care in African healthcare contexts. If causality is confirmed, policy should institutionalize co-creation through shared decision-making protocols and digital health access. Since the co-creation–empowerment link may be weaker for patients with lower education, income, or rural residence, equitable implementation requires targeted health literacy support and outreach to underserved districts. These findings carry practical implications for healthcare policy, management, and practice. By clarifying the complex interplay among these constructs, this research contributes to the literature and informs evidence-based healthcare delivery, with potential to improve patient outcomes and quality of care in African settings.

## Data Availability

The raw data supporting the conclusions of this article will be made available by the authors without undue reservation.
